# Identification of the barrier to gene flow between phylogeographic lineages of the common hamster *Cricetus cricetus*

**DOI:** 10.1007/s13364-012-0075-z

**Published:** 2012-02-28

**Authors:** Agata Banaszek, Joanna Ziomek, Katarzyna A. Jadwiszczak, Ewa Kaczyńska, Paweł Mirski

**Affiliations:** 1 Institute of Biology, University of Białystok, Świerkowa 20B, 15-950 Białystok, Poland; 2 Department of Systematic Zoology, Adam Mickiewicz University, Umultowska 89, 61-614 Poznań, Poland; 3 Mammal Research Institute, Polish Academy of Sciences, ul. Waszkiewicza 1c, 17-230 Białowieża, Poland

**Keywords:** *Cricetus cricetus*, Common hamster, Gene flow, Natural barrier, Habitat composition

## Abstract

In anthropogenically disturbed habitats, natural barriers still exist and have to be recognized, as they are important for conservation measures. Areas of phylogeographic breaks within a species are often stabilized in inhospitable regions which act as natural barriers. An area of contact between phylogeographic lineages of the common hamster (*Cricetus cricetus*) was found in the Małopolska Upland in Poland. A total of 142 common hamsters were captured between 2005 and 2009. All hamsters were genotyped at 17 microsatellite loci and partial sequences of the mitochondrial (mtDNA) control region were obtained. No mixed populations with mtDNA haplotypes of both lineages were found. The distance between marginal populations was about 20 km; no hamsters were found in the area between. A principal components analysis (PCA) was performed on microsatellite data and the greatest change in PC1 scores was found between marginal samples. To define the habitat components responsible for the phylogeographic break, we compared the habitat composition of sites occupied by hamsters with those from which hamsters were absent. We found that hamsters avoided forested areas and sandy soils. The area of the potential barrier was characterized by a high proportion of woodland and unfavorable soils in comparison with neighboring areas inhabited by hamsters. They cannot settle in this area due to their high winter mortality in shallow burrows and high predation in the fields adjacent to forests.

## Introduction

The current distribution of a species and its geographic range limits are the outcome of a balance between several ecological and evolutionary processes (Kirkpatrick and Barton 1997). In the Palearctic, distributions have been mainly shaped by Pleistocene climatic oscillations (Hewitt 1999). Climate changes are also responsible for intraspecies differentiation into phylogeographic lineages. The location of refugia and major geographical barriers to dispersal have shaped the distribution of such lineages within the species. The areas of phylogeographic breaks are very often strongly associated with barriers such as mountain ranges. However, during periods of climate warming, low mountain passes could serve as corridors for dispersal and then the meeting grounds of the lineages would be at the foothills of the mountains. Such areas of contact usually stabilize in regions inhospitable for the species and may clearly reflect its ecological demands. Those low quality areas may be very stable over time and their position would not change until a next major climatic shift (Hewitt 1996, 1999; Swenson and Howard 2005).

The distribution of within-species phylogeographic breaks provides valuable information about the ecology of those species which may be important for management and conservation policies. Today, one of the most pervasive factors limiting the distribution of species is anthropogenic disturbance, which may impact the ranges of species through habitat degradation and loss. Many species have lost large parts of their natural ranges and/or become fragmented. Conservation measures like creating corridors for dispersal require knowledge about barriers to gene flow (Reed and Frankham 2003). Anthropogenic barriers such as roads have been repeatedly shown to slow the dispersal of animals and gene flow (Riley et al. 2006). However, in disturbed habitats, natural barriers still exist and have to be identified.

The common hamster is an ideal example of a European species which has lost large parts of its range; in Central and Western Europe, it presently exists in isolated patches of suitable habitats (Stubbe et al. 1998; Ziomek and Banaszek 2007). It is also special in its choice of habitat, as it lives in Europe almost solely on agricultural land (Nechay 2000). Therefore, it is greatly affected by any anthropogenic changes such as intensification of agricultural practices or urbanization of an area. Natural within-species breaks are already difficult to make out and delineate (Banaszek et al. 2009).

**Fig. 1:**
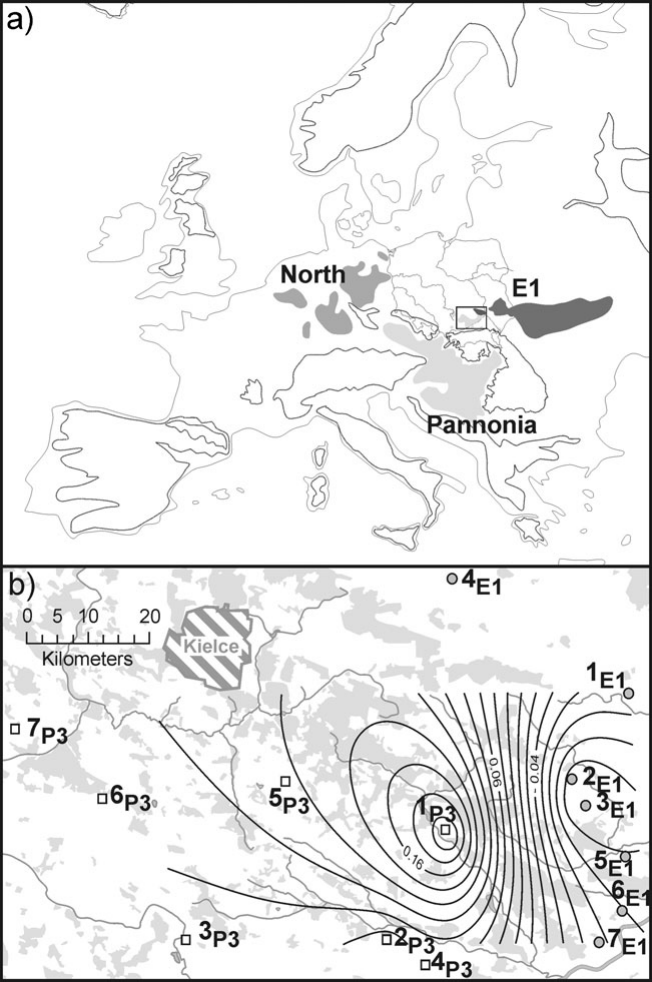
Distribution of common hamster lineages: North, Pannonia and E1 in Europe (**a**) (after Neumann et al. 2005; Banaszek et al. 2010; Banaszek, unpublished data). The distribution of the sampling locations in the Małopolska Upland (**b**). The E1 phylogroup sites are indicated by *gray circles* and P3 (Polish Pannonia) sites by *open squares*. Sampling site labels as in Table 1. The First Principal Component (PC1) scores based on the data set of 17 microsatellite loci in six sampling sites (1_E1_–3_E1_ and 1_P3_–3_P3_) in the Małopolska Upland visualized as isolines superimposed on the study area. The greatest change in PC1 scores indicates the presence of a geographical barrier. Forests are indicated as *gray areas*

The major phylogeographic break in the European common hamster populations seemed to be connected with the Carpathians and German Uplands (Neumann et al. 2005). As hamsters had only eastern refugia in the European steppe zone, their westward migration naturally took two routes: a northern one across the European plains and a southern one to the Carpathian Basin. Three phylogeographic lineages have been described in Europe so far (Fig. 1a): the Pannonia lineage in the Carpathian Basin and southern Poland, the North lineage in Germany and West European countries and the E1 phylogroup in eastern Poland and Ukraine (Neumann et al. 2005; Banaszek et al. 2010, Banaszek, unpubl. data). Net distances (Da) between the phylogroups based on combined mtDNA sequences (the control region, cytb and 16SrRNA) were 1.1% between the North and Pannonia lineages and 0.86% between th E1 and Pannonia ones, while their divergence times were calculated at 85–147 ka and 66–115 ka, respectively (Neumann et al. 2005; Banaszek et al. 2010). The lineages probably separated in the southern Russian and Ukrainian plains before they had recolonized Central Europe. Despite significant differences between the mtDNA lineages and their current allopatry, there are large similarities in their nuclear microsatellites. Still, some differences are visible; for example, between E1 and Polish Pannonia (P3), there is a small (3.5%) but statistically significant proportion of between-group variation revealed by analysis of the molecular variance (AMOVA) (Banaszek et al. 2011).

So far, the only known contact site of the phylogeographic lineages of the common hamster has been found in Poland. The E1 lineage inhabits the Lublin Upland with Roztocze and the northern part of the Małopolska Upland. The P3 lineage occurs in the southern part of the Małopolska Upland and some isolated areas of the Krakow-Częstochowa Upland, Upper Silesia and the Sandomierz Basin (Banaszek et al. 2010). Judging from the present-day distribution of the lineages in Europe, the Pannonia lineage entered the Małopolska Upland from the south and E1 from the north. In the Małopolska Upland, the lineages could form mixed populations or establish a boundary along some ecological barrier. We supposed that a belt of poorer soils could serve as such (Banaszek et al. 2009). To confirm our hypothesis, we planned work with the following aims:

1. To describe the distribution of the phylogeographic lineages in the Małopolska Upland;
2. To test for the presence of a significant population structure in this area and to identify a potential barrier to gene flow and
3. To describe habitat features of the potential barrier area.

## Material and methods

### Sample collection for genetic analyses

A total of 142 common hamsters were captured in live traps set near their burrows during the years 2005–2009 in the Małopolska Upland (Table 1). The hamsters were trapped in six sites, 1_E1_, 2_E1_, 3_E1_, 1_P3_, 2_P3_ and 3_P3_, forming roughly a 74.6 km transect through the study area (Fig. 1b). In these sites, larger samples were collected (Table 1). The samples 1_E1_ and 3_P3_ were used to describe the mtDNA and microsatellite diversity in the Polish part of the common hamster range (samples M1 and M2 in Banaszek et al. 2009, 2010, 2011). Once the contact zone between phylogeographic groups in the Małopolska Upland had been identified, we searched for hamsters in numerous localities to check for the direction of the contact zone in a larger area. One to three hamsters were collected in eight sites (Table 1, Fig. 1b).

**Table 1:** Sampling locations and the number of common hamsters used for genetic analysis. The locations are assigned to a phylogroup on the basis of the *ctr* mtDNA haplotype. Haplotype diversity (*h*) and nucleotide diversity (*π*) of populations are indicated

Phylogroup	Sample	Number	Year of trapping	Haplotypes	*h*	*π* (%)
E1	1_E1_	38	2005, 2006	Ccdl36	–	–
	2_E1_	14	2008	Ccdl36	–	–
	3_E1_	7	2008	Ccdl36	–	–
	4_E1_	1	2009	Ccdl36	–	–
	5_E1_	1	2009	Ccdl36	–	–
	6_E1_	3	2009	Ccdl36	–	–
	7_E1_	1	2009	Ccdl36	–	–
Total E1 65				Ccdl36 (1.0)		
P3	1_P3_	22	2008	Po2 (0.95), Po5 (0.05)	0.091 ± 0.081	0.025 ± 0.051
	2_P3_	21	2008	Po2 (0.95), Po5 (0.05)	0.125 ± 0.106	0.034 ± 0.062
	3_P3_	29	2005	Po2	–	–
	4_P3_	1	2009	Po2	–	–
	5_P3_	2	2009	Po2 (0.5), Po5 (0.5)	1.0 ± 0.5	0.273 ± 0.386
	6_P3_	1	2009	Po2	–	–
	7_P3_	1	2009	Po4	–	–
Total P3 77				Po2 (0.95), Po4 (0.01), Po5 (0.04)	0.111 ± 0.050	0.030 ± 0.056

The captured hamsters were put under anesthesia, and their ear tips were clipped. All the animals were released. The capture and handling of animals in the field was conducted under the permissions of the Minister of the Environment DOPog.-402-02-54/04/aj, DLOPiK-op/ogiz-4200/IV-3/815/08/aj and DKFOPogiz-4200/IV-18/1117/08/ls Warsaw, Poland and the Local Ethics Committee on Animal Research in Białystok 2003/53, 2007/69 and 2008/38 Białystok, Poland.

### DNA extraction and genotyping

Total genomic DNA was extracted from frozen ear tips using the GenomicMini kit (A&A Biotechnology). All hamsters were genotyped at 17 microsatellite loci developed for the common hamster: Ccrμ3, 4, 10, 11, 12, 13, 15, 17, 19 and 20 (Neumann and Jansman 2004, AJ532553–AJ532554, AJ532556–AJ532563) and CriCriIPK-01, -03, -05, -06, -07, -09 and -12 (Jakob and Mammen 2006, AM167541, AM167543–AM167548). Partial sequences of the mitochondrial control region (*ctr*) were also obtained from all animals. The PCR profiles for microsatellite and *ctr* amplification, the method of microsatellite analysis, and sequencing reactions for *ctr* were performed as described previously (Banaszek et al. 2009, 2010, 2011).

### Genetic analysis

Unless indicated otherwise, all the following analyses were performed on the six larger samples, excluding all the sites where single individuals were collected. To test our data for genotyping errors such as large allele dropout, the presence of stutter peaks, or null alleles, we used Micro-Checker (van Oosterhout et al. 2004). This software discriminates between Hardy–Weinberg deviations caused by lack of panmixia, which may be common in endangered species and the effects of genotyping errors. We found no evidence for genotyping errors including null alleles. Linkage disequilibria were computed in Arlequin 3.5.1.3 (Excoffier and Lischer 2010); none were found to occur.

We used Structure 2.3.3 (Pritchard et al. 2000) to test if the samples originated from separate populations. The program calculates a maximum likelihood ratio so as to assign individuals to their most probable source population. The clustering model does not use any a priori information about the populations from which individuals were sampled but creates populations minimizing linkage and Hardy–Weinberg disequilibria. In the first analysis, we checked if the six larger samples formed genetic clusters. We performed ten independent runs checking the number of subpopulations (between *K* = 1 and *K* = 8) with 500,000 iterations and a 50,000 burn-in periods. The admixture model and correlated allele frequencies were used in simulations. The admixture model represents populations which have recently or currently had enough gene flow for individuals to have ancestors in more than one population. The correlated allele frequencies model assumes that the populations originated from a common ancestor population in the past and the differences between them are the result of drift that has occurred since that time. Populations that have diverged not long ago have more similar allele frequencies. Although presently, the gene flow between common hamster populations might be lower considering the population crash, this problem is quite recent (Banaszek et al. 2011). However, even very low levels of current gene flow would result in some admixed individuals and a correlation in allele frequencies. Another problem is the contact zone in the area. The phylogroups in contact here are ancient (Neumann et al. 2005; Banaszek et al. 2010), and no admixed individuals between lineages were found in a countrywide microsatellite research (Banaszek et al. 2011). However, as we aim to establish the strength of the barrier between lineages, it would be inappropriate to choose a no admixture model and independent allele frequencies, which would assume a priori a complete lack of gene flow and total independence between the lineages. The best-fit model of the number of genetic populations was chosen on the basis of the *K* statistics of Evanno et al. (2005), based on the rate of change in the log probability of data between successive *K* values.

In the next analysis, we used all the individuals collected in the study area and checked to which genetic cluster single individuals should be assigned. The Structure program was also employed to identify migrants or the offspring of migrants with the same model parameters as for the clustering option. In genetic assignment tests, the a priori information about the sampling sites is given. Structure assigns to each individual the probability of origin from each sampled location, assuming that the population of origin was sampled. We used a limit of 0.9 of the probability value (*q*) for origin population assignment (Manel et al. 2002). We also conducted assignment tests using GeneClass2 (Piry et al. 2004). This software does not assume that all the true populations were sampled. The program uses allele frequencies within populations to compute the likelihood of an individual genotype occurring in each population and then compares the likelihoods against simulated genotypes to provide a ‘probability of belonging’ (*P*) for each individual from each sampled population (Paetkau et al. 1995; Manel et al. 2002). We used the frequency-based method of Paetkau et al. (1995). Individual genotypes were compared against 10,000 simulated genotypes (Paetkau et al. 2004).

Arlequin3.5.1.3 software was used to calculate the basic genetic variability indices, i.e., the mean number of alleles (A) and heterozygosities (Ho and He). The tests for departures from the Hardy–Weinberg equilibrium for the populations were also computed in Arlequin. The Fis value based on Weir and Cockerham (1984) was calculated and tested with permutations using FSTAT 2.9.3 (Goudet 1995). For multiple comparisons, we used the Bonferroni correction wherever applicable.

Population differentiation based on Fst values between pairs of populations was calculated in Arlequin. Statistical significance was estimated with 10,000 permutations. The model of isolation by distance (IBD) was tested in Isolde implemented in Genepop (Raymond and Rousset 1995). Geographic straight line distances between the sampling sites were measured with a distance tool in ArcView 3.1.2 (ESRI).

Principal components analysis (PCA) was performed on microsatellite data in GenAlEx 6.0 (Peakall and Smouse 2006). The first axis of the PCA scores was used to delineate the barrier to gene flow among the sampling sites in the Małopolska Upland. Contour lines were interpolated using the kriging algorithm and superimposed on a geographic map of the study area (Fig. 1b) using Surge software (http://www.geocities.com/miroslavdressler/surgemain.htm). Barriers were identified as zones of maximum slope following the contour lines of the PC1 scores. PCA analysis was also used to visualize the population differentiation in the area of contact and to get additional support information for the number of genetic populations established by Structure.

### Habitat analysis

**Fig. 2:**
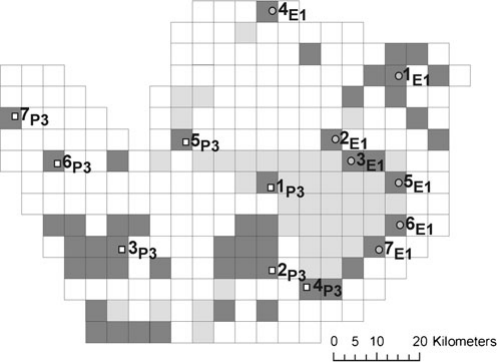
A 5 × 5 km grid of squares superimposed on the study area in the Małopolska Upland for common hamster habitat analysis. An *empty square* = no data on hamster occurrence, *dark gray* = an occupied square, *light gray* = a nonoccupied square. Sampling site labels as in Table 1 and Fig. 1b

The area in which hamsters were collected was covered with a grid of 5 km × 5 km squares (Fig. 2). The squares which represented Kielce City and the urbanized area around it were excluded from the analysis, as the absence of hamsters there did not reflect their habitat preferences (Figs. 1b and 2). We were able to identify 51 squares occupied by hamsters and 49 not occupied out of the total of 249 squares. For this analysis, we used all our data on the common hamster occurrence since 2000 (Ziomek and Banaszek 2007; this paper). The habitat features of the occupied versus nonoccupied squares were compared in terms of soil classes and the type of vegetation cover. The information about the soils was taken from an agricultural soil map (Institute of Soil Science and Plant Cultivation, State Research Institute) analyzed in ArcGIS 10.0. Three types of soil differing mainly in genesis were used in the analyses: loesses, sands and clays as well as five forms distinguished on the basis of morphology: chernozems, brown earths, rendzinas, sandy earths and silts (peat soils were excluded due to their very low proportion in the analyzed area). Using CORINE (Coordination of Information on the Environmnent) land cover maps (2005), we distinguished four main categories of habitat: 1) arable land; 2) forests; 3) grassland, meadows and pastures and 4) mosaics of small gardens, pastures, fallow land and natural vegetation. Other habitats were excluded from the analysis. Each occupied or nonoccupied square was characterized as to the proportion of soil classes and land cover. Differences between the occupied and nonoccupied squares were tested by the bootstrap *t* test with equal variances not assumed (10,000 bootstrap) in Rundom Pro 3.14 (Jadwiszczak 2009). The test is a randomization equivalent of Student's *t* test.

Once the habitat features most important for the common hamster were identified, the barrier area could be described. It consisted of 24 squares between the marginal points of the phylogeographic groups, which were thoroughly checked for the presence of hamsters in 2009 and 2010 (Fig. 2).

## Results

Three hundred and sixty-six base pairs of the mitochondrial control region were sequenced or all hamsters. Four previously described haplotypes: Ccdl36 (Neumann et al. 2004, GenBank accession no. AJ 633738), Po2, Po4 and Po5 (Banaszek et al. 2009, EU 016107, EU 016109, EU 016110), characteristic of either the E1 or the P3 phylogroup (Banaszek et al. 2009, 2010), were discovered in the study area (Table 1). Haplotype Ccdl36 was found in its northeastern part where the E1 phylogroup was expected. There was no variability in *ctr* in this region. In the southwestern part, haplotype Po2 predominated, as expected for the P3 lineage in the Małopolska Upland. Haplotype Po4 was found in a single hamster collected in the westernmost site 7_P3_. There were also three individuals with the Po5 haplotype, which means that three populations (1_P3_, 2_P3_ and 5_P3_) were polymorphic with Po2 and Po5 haplotypes. However, the nucleotide diversity value for the total P3 lineage in the Małopolska Upland is very low (Table 1). No mixed populations with haplotypes characteristic of both lineages were found. Moreover, the single individuals collected from sites 4_E1_–7_E1_ and 4_P3_–7_P3_ had the haplotypes expected of their geographical location: sites 4_E1_–7_E1_ of the E1 lineage and sites 4_P3_–7_P3_ of the P3 lineage. The distance between the marginal E1 and P3 sampling sites is smaller than 20 km, for example, sites 3_E1_ and 1_P3_ are 18.8 km apart and sites 7_E1_ and 4_P3_ located in the Vistula valley are 18.1 km apart (Fig. 1b). The area separating the marginal populations was thoroughly checked for the presence of hamsters, and none were found. The fields following approximately a straight line between 3_E1_ and 1_P3_ were checked twice in 2009 and 2010. The remaining areas between the marginal points of the lineages, indicated in Fig. 2, were checked for hamsters in 2010. The squares between the 7_E1_ and 4_P3_ sites marked as occupied by hamsters (Fig. 2) were inhabited in 2001 (Ziomek and Banaszek 2007). In the years 2009–2010, the Vistula River flooded heavily, and these populations are now absent.

**Fig. 3:**
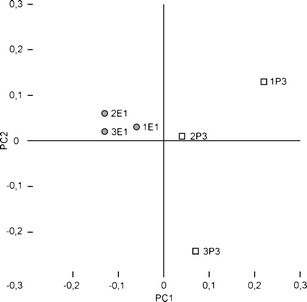
Differentiation of the common hamster populations in the Małopolska Upland as shown by the Principal Components Analysis (PCA) based on the data set of 17 microsatellite loci in six sampling sites (1_E1_–3_E1_ and 1_P3_–3_P3_) in that region. The scores shown are those on the first and second principal coordinates. Sampling site labels as in Table 1 and Fig. 1b

Seventeen microsatellite loci were genotyped for all animals. First, we checked if our samples formed genetic clusters. For the Structure clustering model, we used six samples of seven to 38 hamsters. The *K* statistics of Evanno et al. (2005) took the maximum value for *K* = 2. One genetic cluster was formed by 3_P3_ sample and the other by all the other samples. However, we suppose that the Structure algorithm did not find a real biological structure at this level, although at first sight *K* = 2 would correspond to two phylogeographic lineages making contact in this area. As Structure just separates one sample, it probably attempts to minimize the disequilibria, excluding the 3_P3_ sample which is inbred and not in HW equilibrium (see further sections of “Results”). Moreover, the *K* value did not plateau at higher numbers of genetic clusters but reached another peak at *K* = 4. One genetic cluster is formed by the E1 lineage samples, while the three samples from the P3 group formed independent genetic clusters (Table 2). The proportion of individuals assigned to the cluster was very high and invariably exceeded 0.9. PCA analysis also showed a stronger clustering of the samples of the E1 lineage and a more scattered picture for the P3 samples, which shows their greater differentiation (Fig. 3). The PCA results are consistent with the conclusion of *K* = 4 in this area supplied by the Structure clustering method.

**Table 2:** Average proportion of membership of each sample in four genetic clusters identified by Structure. The assignment with probability value higher than 0.9 indicated by italics

Phylogroup	Sample	Genetic cluster			
		1_E1_ + 2_E1_ + 3_E1_	1_P3_	2_P3_	3_P3_
E1	1_E1_	*0.982*	0.004	0.007	0.006
	2_E1_	*0.983*	0.003	0.008	0.007
	3_E1_	*0.948*	0.005	0.043	0.003
	4_E1_	0.822	0.003	0.174	0.002
	5_E1_	*0.949*	0.032	0.008	0.011
	6_E1_	0.897	0.007	0.083	0.013
	7_E1_	*0.931*	0.003	0.003	0.063
P3	1_P3_	0.015	*0.963*	0.011	0.011
	2_P3_	0.008	0.003	*0.986*	0.002
	3_P3_	0.010	0.003	0.018	*0.969*
	4_P3_	0.013	0.004	*0.956*	0.027
	5_P3_	0.004	0.581	0.006	0.409
	6_P3_	0.004	0.037	0.716	0.243
	7_P3_	0.003	0.006	*0.986*	0.004

The Structure assignment tests did not identify any individual as first-generation migrants. The same result was given by GeneClass2. One hamster from the 3_P3_ population could be a descendant of migrating animals from the 2_P3_ population, as indicated by Structure. No migrants between the populations of the phylogroups were found. The Structure clustering model was also used to assign hamsters from the 4_E1_–7_E1_ and 4_P3_–7_P3_ sites to the identified genetic clusters (Table 2). The animals from 4_E1_–7_E1_ could be assigned to the E1 cluster, although for hamsters 4_E1_ and 6_E1_, the probability values were slightly lower than 0.9. Hamsters from the 4_P3_–7_P3_ sites could be assigned to the P3 genetic clusters. Those from the 5_P3_ and 6_P3_ sites could not be assigned to one genetic cluster, but in both cases, two genetic clusters with higher probabilities of assignment were of the P3 lineage (Table 2). In general, hamsters from the 4_E1_–7_E1_ and 4_P3_–7_P3_ sites were assigned as could be expected from their membership in the mtDNA lineages (Table 1).

The mean number of alleles for the analyzed clusters is the highest for the E1 cluster and the lowest for the 1_P3_ population (Table 3). Population heterozygosities are moderate, and again, for 1_P3_, the values are the lowest. However, there are no clear differences between the observed and the expected heterozygosities for the populations, which could indicate random mating. This is supported by low Fis values. Only the 3_P3_ population is inbred. This sample and 2_P3_ are also in HW disequilibrium.

**Table 3:** Genetic diversity measures for the genetic clusters identified in the Małopolska Upland samples of the common hamster. Mean number of alleles (A), observed (Ho) and expected (He) heterozygosity, inbreeding coefficient (Fis) and probability for HW equilibrium test are given. Statistical significance of Fis or HW disequilibrium is indicated by an asterisk

Phylogroup	Genetic cluster	Number	A	Ho	He	Fis	HW equilibrium
E1	1_E1_ + 2_E1_ + 3_E1_	59	7.56	0.686	0.723	0.064	0.023
P3	1_P3_	22	3.41	0.465	0.499	0.070	0.239
	2_P3_	21	5.59	0.696	0.700	0.008	0.002^*^
	3_P3_	29	4.94	0.538	0.600	0.106^*^	0.003^*^

Pairwise Fst values between populations ranged from 0.14 to 0.35, and all the estimates were statistically significant. The Fst values between pairs of populations of different phylogroups was over 0.2, save cluster 2_P3_, which had moderate Fst values with that of the E1 lineage (Table 4). The Fst value for the clusters in the P3 lineage was invariably high. No correlation (*r* = 0.21, *p* = 0.18) between the genetic (Fst/1-Fst) and the geographic distance was found.

**Table 4:** Pairwise comparison of Fst values between genetic clusters of the common hamster in the Małopolska Upland. All comparisons are statistically significant

Genetic cluster	1_E1_ + 2_E1_ + 3_E1_	1_P3_	2_P3_
1_P3_	0.236		
2_P3_	0.140	0.202	
3_P3_	0.270	0.354	0.255

The first and second PCA axes explained 38.61% and 32.51%, respectively, of the total variability at microsatellite loci. A synthetic genetic map for the first axis of PCA scores was superimposed on a geographic map of the study area in Fig. 1b. The change in PC1 scores is the greatest between samples of different phylogeographic lineages, i.e., 2_E1_ + 3_E1_ and 2_P3_ (the maximum slope following the contour lines), suggesting that there is a barrier to gene flow in this area. This high gradient region of genetic change closely corresponds to the area where no hamsters were found in two consecutive years.

The differences in habitat composition between the occupied and the nonoccupied squares are given in Table 5. We found six factors differentiating highly significantly the two types of squares: loesses, chernozems and arable land were preferred by hamsters, while sands or sandy soils and forests were avoided. There were also significant differences in the share of brown earths, which were more frequent in the occupied squares. Moreover, the occupied squares showed a slightly higher proportion of silts, and the difference between the occupied and the nonoccupied squares was marginally significant. Other habitat factors were neutral for the common hamster (Table 5). The barrier area, which comprised 24 squares where no hamsters were found, was described in terms of factors significantly differentiating the occupied from the nonoccupied squares (Table 6). Sands and sandy soils prevailed there, while the proportion of loesses, chernozems, brown earths and silts was very low. Arable land and forests had a similar share.

**Table 5:** Habitat selection by the common hamster in the Małopolska Upland. The proportion of soil types, soil forms and land cover in occupied (*n* = 51) and unoccupied squares (*n* = 41) were compared by the bootstrap *t* test. Background (total number of squares = 249) composition is also given

Habitat feature		Squares			Test value and probability	Selection
		Occupied	Unoccupied	Total		
Soil types	Loess	0.43	0.10	0.27	4.36, 0.0001	Preferred
	Sand	0.27	0.64	0.34	−5.38, 0.0001	Avoided
	Clay	0.06	0.13	0.14	−1.69, 0.10	Neutral
Soil forms	Sandy earths	0.32	0.75	0.49	−6.35, 0.0001	Avoided
	Chernozem	0.26	0.05	0.08	2.90, 0.007	Preferred
	Brown earths	0.20	0.08	0.28	2.27, 0.029	Slightly preferred
	Silt	0.14	0.04	0.12	2.14, 0.052	Slightly preferred
	Rendzina	0.08	0.03	0.04	1.46, 0.157	Neutral
Land cover	Arable land	0.63	0.42	0.53	6.23, 0.0001	Preferred
	Forest	0.09	0.33	0.19	−6.63, 0.0001	Avoided
	Mosaic	0.15	0.14	0.16	1.42, 0.163	Neutral
	Pastures	0.09	0.08	0.09	1.02, 0.312	Neutral

**Table 6:** Habitat composition of the barrier area (24 squares) from which the common hamsters were absent. Habitat features shown are only those with a significant difference (given in Table 5) between the occupied and the non-occupied squares

Habitat feature		Mean	Min	Max	SE
Soil types	loess	0.05	0	0.57	0.03
	sand	0.75	0.32	1.00	0.04
Soil forms	sandy earths	0.84	0.38	1.00	0.04
	chernozem	0.02	0	0.51	0.02
	brown earths	0.05	0	0.62	0.03
	silt	0.03	0	0.35	0.01
Land cover	arable land	0.40	0.03	0.70	0.03
	forest	0.42	0.08	1.00	0.05

## Discussion

An area that forms a barrier between phylogeographic lineages of the common hamster was found in the Małopolska Upland. Although the time of contact in this area is not known, we would like to stress that it is unlikely to be connected with the recent population crash. On the distribution map of the common hamster in Poland created by Surdacki (1971) and based on the research performed during the 1960s when hamsters were abundant, a gap in the hamster distribution equivalent to the barrier area described here is visible too.

The lineages could come into contact here not earlier than the end of the Last Glaciation, as hamsters of the E1 lineage appeared in the Małopolska Upland quite late following the demographic expansion of this group in southeastern Poland, which was dated roughly to the end of the Last Glaciation and the beginning of the Neolithic (Banaszek et al. 2010). E1 hamsters could not move into the area of Poland, as the eastern route of migration was probably blocked by the harsh conditions on the Lublin Upland throughout the Vistulian. The Neolithic spread of agriculture resulted in high densities of hamster populations, which triggered long migration movements, and at that time, the Vistula River might have been crossed by hamsters of the E1 lineage. In turn, by paleontological and molecular data, the Pannonia lineage (P3) had already been present in southern Poland for quite a long time as it appeared there in the Middle Vistulian V2 (53.35 ka) (Kowalski 2001; Banaszek et al. 2010). It is not possible to establish currently if P3 hamsters were present in the northern part of the Małopolska Upland before the E1 lineage inhabited this area. The distribution of the P3 lineage might have been wider, but for some reasons, they might have become extinct and the area could then be settled by the E1 lineage. On the other hand, during the Last Glaciation, loess sediments gradually formed in the Małopolska Upland (Lindner and Marks 2008). In general, migration between smaller loess patches in the southern and northern parts of the upland could have been impeded to a greater extent than today. Hence, it is also possible that hamsters of the P3 lineage never extended their distribution northwards. In this case, the current barrier area could be very long lasting, although the habitat features that stopped the northward spread of hamsters might not have been precisely the same as today.

On the evidence given by local farmers, in some years, hamsters appear and try to colonize at least the edges of the barrier area. We suppose that they cannot survive for long in this region. They try to settle in seemingly continuous habitats, but their survival must be decidedly lower than in the surrounding areas. The two main causes of their mortality are predation and death during hibernation due to various factors connected with the amount of food stored and burrow traits. For example, the ability to winter depends on the depth of burrows (Nechay 2000). The barrier area is mostly composed of sandy soils in which stable, deep burrows cannot be built. Moreover, the high percentage of woodland in the area indicates increased predation risk. Although fields contribute a similar proportion as forests there, they are usually smaller than in more open terrain and generally neighbor on forests. Hamsters do not inhabit forests, and they avoid fields located close to their edges (Nechay 2000), as many birds of prey nest there and use the surrounding open area for feeding (Zub et al. 2010). Summing up, habitat quality in the barrier area is too low for hamsters. On the other hand, it appears that the distance between favorable habitat patches is too great to cross and does not lie within dispersal abilities of single individuals, i.e., there is some threshold distance of unsuitable habitats which serves as an efficient barrier. The distance between the marginal populations that differ by mtDNA *ctr* haplotypes is less than 20 km.

The diversity levels of the hamster populations in the Małopolska Upland are quite typical of the Polish part of the species' range (Banaszek et al. 2009, 2011). The *ctr* mtDNA diversity is absent or low, and microsatellite diversity is moderate with fairly high heterozygosity levels. The presence of just one *ctr* haplotype in the E1 population reflects its recent origin (Banaszek et al. 2009, 2011), while the Pannonian populations show some *ctr* diversity, which is in agreement with their longer history in this area. The 1_P3_ population which forms the northern border of the Pannonia lineage here shows the lowest microsatellite diversity levels, visibly lowered in comparison with the other populations. This population lives in a small patch of high quality habitat, i.e., on a loess hill. However, this area seems unstable, with abrupt elevation changes and steep slopes. In 2009, after heavy rains, its hamster population was almost eradicated. Probably, 1_P3_ is a typical sink population which existence depends on migration from other Pannonian populations in this area which serves as a source (Runge et al. 2006). In years with a higher density, an influx of hamsters saves the population. This hypothesis remains to be tested with possibly more samples from the P3 lineage and additional data on the reproduction and survival of hamsters in these populations.

The gene flow between hamster populations is difficult to estimate. The pairwise Fst values between populations are usually quite high and statistically significant (Neumann et al. 2005; Banaszek et al. 2011). Hamsters, at least females, are quite sedentary (Weinhold 1996), and the IBD pattern of population differentiation is usually not found (Neumann et al. 2005; Banaszek et al. 2011), which is also the case in the study area. With low dispersal and high reproductive output, the local populations of the common hamster form very close genetic clusters, as was shown by the Structure clustering method. Moreover, no first-generation migrants were found in this area by either Structure or GeneClass2 algorithms. As currently populations rarely reach high densities, long distance migrations may be quite rare. However, in the northern part of the Małopolska Upland, Structure found one genetic population, which may reflect a greater genetic similarity of the population on the leading edge of the migration wave (Hewitt 1996; Hampe and Petit 2005), or more opportunities for gene flow in this area. The P3 samples, on the other hand, are separate genetic clusters, and gene flow between them seems to be restricted. A more important question is if there is any gene flow across the barrier. On the one hand, the gene flow, if present, is restricted, as shown by the Fst values and the results of assignment analysis where the Structure and GeneClass algorithms did not find any first-generation hamsters. Moreover, single hamsters (4_E1_–7_E1_ and 4_P3_–7_P3_ sites), although not always assigned with a high probability to one particular population, were always assigned to the expected phylogroup. On the other hand, there is an obvious similarity in microsatellite diversity between the phylogeographic lineages. The difference in microsatellite diversity between the phylogroups found by AMOVA, although significant, was very small, about 3.5% (Banaszek et al. 2011). In general, a similarity in nuclear markers may reflect gene flow between lineages and, to some extent, homoplasy: the allelic composition of the loci, mutating mostly by the stepwise mutation model (SMM), has to be similar. Currently, the lineages of the common hamster are monophyletic in mtDNA with no mixed populations (Neumann et al. 2005; Banaszek et al. 2010). A possible explanation for lack of mixed mtDNA populations with a high similarity of the nuclear markers is male migration with females rarely taking part in dispersal. In hamsters, males are the migrating sex, as was shown for *Tscherskia triton* (Song et al. 2005) and also suggested for *Cricetus cricetus* on the basis of relatedness analysis (Banaszek and Ziomek 2012). Populations with mixed mtDNA haplotypes could become mtDNA pure quite quickly through lineage sorting (McCracken and Sorenson 2005). Rapid loss of haplotypes is quite possible in common hamster populations which are naturally often bottlenecked (Neumann et al. 2004, 2005). In the Małopolska Upland, there may be some male-mediated gene flow across the barrier. The most probable corridor for migration in this area is along the Vistula River (Fig. 1b). Hamsters readily settle along rivers where agriculture is usually well developed on rich silt soils. Since 2000, hamster localities have been found in most squares along the Vistula River (Fig. 2, Ziomek and Banaszek 2007). However, the Vistula is not fully regulated and floods quite regularly. In 2010, most of the riverside was inundated and hamster populations were largely eliminated from this area. Females, as more sedentary, do not cross the barrier even along the river in the periods between floods, or cross it very rarely. The lowered Fst values between the 2_P3_ and E1 populations in comparison with the values for other population pairs may indicate this opportunity of gene flow.

In conclusion, spatial and temporal variations in habitat quality serve as efficient barriers between the phylogeographic lineages of the common hamster in the Małopolska Upland. The strength of such a seemingly insubstantial barrier is an important clue for any conservation plans such as creating corridors for migration or agroreserves for hamster protection. It is important that any such conservation plans should be preceded by a scrupulous investigation of habitat quality. Placing agroreserves in sink habitats would result in a waste of efforts and funds and a population declining to extinction in low density years. Similarly, corridors not taking into account small natural barriers will result in no gene flow or, at most, some male dispersal with females effectively isolated.
